# Volumetric capnography: lessons from the past and current clinical applications

**DOI:** 10.1186/s13054-016-1377-3

**Published:** 2016-06-23

**Authors:** Sara Verscheure, Paul B. Massion, Franck Verschuren, Pierre Damas, Sheldon Magder

**Affiliations:** Department of Critical Care Medicine, University of McGill, Montreal, Quebec Canada; Department of General Intensive Care, University Hospital of Liege, Liege, Belgium; Department of Emergency Medicine, Cliniques universitaire Saint-Luc, Université Catholique de Louvain, Brussels, Belgium

## Abstract

Dead space is an important component of ventilation–perfusion abnormalities. Measurement of dead space has diagnostic, prognostic and therapeutic applications. In the intensive care unit (ICU) dead space measurement can be used to guide therapy for patients with acute respiratory distress syndrome (ARDS); in the emergency department it can guide thrombolytic therapy for pulmonary embolism; in peri-operative patients it can indicate the success of recruitment maneuvers. A newly available technique called volumetric capnography (Vcap) allows measurement of physiological and alveolar dead space on a regular basis at the bedside. We discuss the components of dead space, explain important differences between the Bohr and Enghoff approaches, discuss the clinical significance of arterial to end-tidal CO_2_ gradient and finally summarize potential clinical indications for Vcap measurements in the emergency room, operating room and ICU.

## Background

Ventilation dead space (VD) refers to the parts of the lung and airways that do not partake in the clearance of carbon dioxide (CO_2_) and indicates the inefficient portion of ventilation. When CO_2_ production and total ventilation (VE) are constant, arterial PCO_2_ (partial pressure of carbon dioxide) increases in proportion to the increase in VD. Capnography is the measurement of expired PCO_2_. Time-based capnography refers to the elimination of CO_2_ over time and gives an indication of ventilation inefficiency. Expired CO_2_ can be obtained by sampling either mainstream or side-stream expiratory flow. In the mainstream approach the infrared light source and sensor are placed in the primary airflow tube so that expired gas is sampled directly during expiration and the CO_2_ signal is in-phase with the air-flow and pressure signals. In the side-stream technique gas is continuously aspirated from the primary airway through a sampling line that is placed between the patient and the Y-piece of the ventilator. This creates a slight delay between collection and gas analysis [1].

Although simple to apply, standard time-based capnography does not allow identification of the volume components of the signal, which is necessary for determination of the anatomical source of CO_2_ and understanding the pathological processes. Separation of the components requires simultaneous measurement of volume and CO_2_ by what is called volumetric capnography (Vcap). In this technique expired CO_2_ is plotted against exhaled lung volume. This allows breath-by-breath quantification of the volume of lung units that are ventilated but not perfused and measurement of alveolar VD. The rationale for the analysis is similar to that of the nitrogen (N_2_) washout approach developed by Fowler [2] and later further developed by Fletcher and colleagues in the early 1980s [3]. Newer generation ventilators such as Hamilton-T1, Dräger Evita XL, and Maquet Servo-I, have integrated mainstream “volumetric” CO_2_ sensors that allow calculations of mixed expired CO_2_ pressure (PĒCO_2_) and real-time VD fraction [4, 5]. However, these devices do not process the breath-by-breath volume signal to perform Vcap; this requires data processing software to relate the CO_2_ signal to volume signal. Examples are NICO_2_® capnograph (Respironics, Wallingford, Connecticut), CO_2_-SMO® capnograph (Novametrix, Wallingford, Connecticut) and S/5-COLLECT (Datex-Ohmeda, GE Healthcare, Helsinki, Finland).

## Definitions

**Fig. 1:**
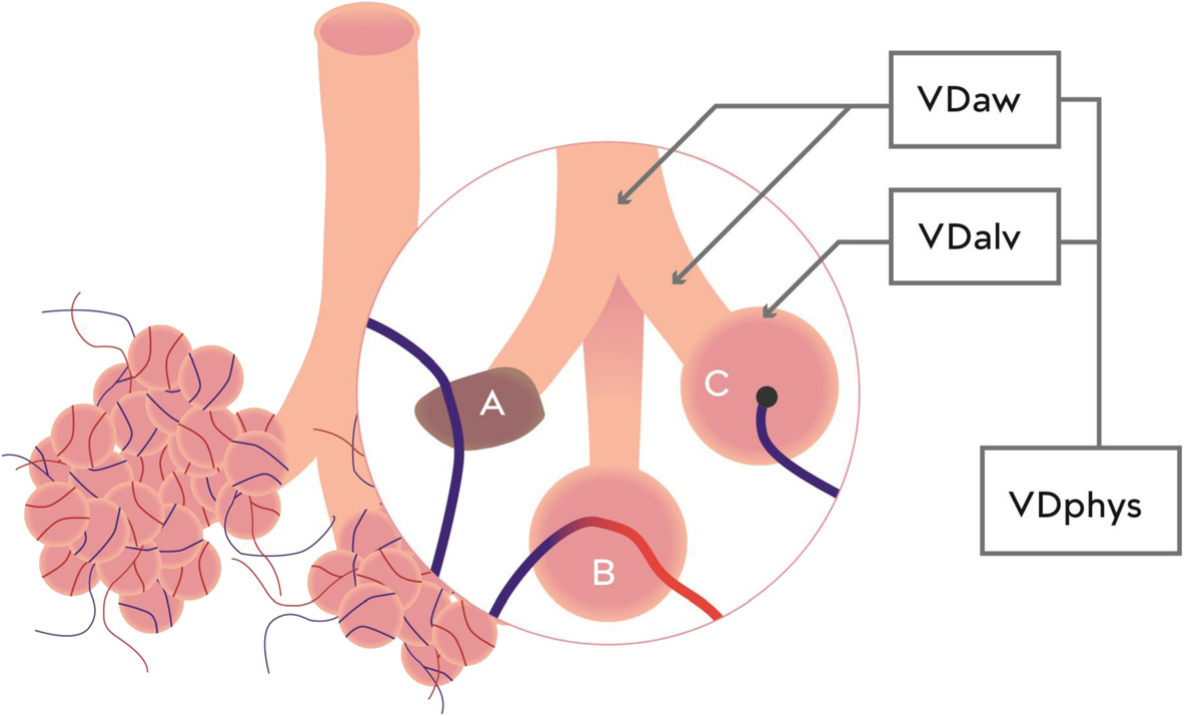
Riley three compartment model. Compartment A: shunt = perfused but not ventilated alveolae (V/Q = 0). Compartment B: ideal condition. Compartment C: dead space = ventilated but not perfused alveolae (V/Q = ∞). *VDaw* airway dead space, *VDalv* alveolar dead space, *VDphys* the sum of airway and alveolar dead space

The terms physiological or respiratory dead space (VDphys) refer to lung units that are ventilated but do not contribute to gas exchange because the expired gas from these units has no contact with pulmonary capillary blood flow. VDphys can be divided further into alveolar dead space (VDalv) and anatomical dead space, which also is known as airway dead space (VDaw) [6, 7]. As shown in Fig. 1, VDaw corresponds to the volume in conducting airways and ends at the alveolar compartment. The term VDalv refers to volume in alveoli that is ventilated but not perfused (compartment C in Fig. 1) as described by Riley and Cournand [8]. This compartment corresponds to West zone I as identified by the multiple inert gas elimination technique [9]. West zone I occurs when alveolar pressure is greater than the pressure inside the collapsible pulmonary vessels and the alveolar pressure thus stops pulmonary flow in that region. This can occur with hyperinflation of the lungs or even due to gravitational effects as vascular pressures in upper regions become less than alveolar pressure.

In the clinical context, VDalv typically arises through two general processes. The first involves over-inflation of the lung, which can be due to dynamic hyperinflation because the expiratory period is too short to completely expire the inflated volume, the level of positive end-expiratory pressure (PEEP) is high, or the delivered tidal volume is large. The second process involves decreased pulmonary perfusion or changes in the distribution of perfusion caused by either direct obstruction of arterial pulmonary vessels or by reduction of output from the right ventricle. VDaw normally is relatively fixed, but it changes with changes in body position or tracheal diameter. The latter can be decreased by the presence of an endotracheal tube and increased by high levels of PEEP [10]. Additional equipment attached in series between the patient’s mouth and the Y-piece of the ventilator circuit also increases VDaw; this is called instrumental dead space (VDinst). The product of VDphys (mL) and respiratory rate give the dead space ventilation (VD) (L/min).

VE is the sum of alveolar ventilation (VA) and physiological VD and is given by [4]:

1 $$ \mathrm{V}\mathrm{E} = \mathrm{V}\mathrm{A} + \mathrm{V}\mathrm{D}\ \left(\mathrm{L}/ \min \right) $$

In 1891, Bohr proposed an equation to calculate physiological dead space normalized to tidal volume (VT):

2 $$ \mathrm{VDphys}/\mathrm{V}\mathrm{T} = \left(\mathrm{F}\mathrm{A}\mathrm{C}{\mathrm{O}}_2\hbox{-}\ \mathrm{F}\bar{\mathrm{E}} \mathrm{C}{\mathrm{O}}_2\right)/\mathrm{F}\mathrm{A}\mathrm{C}{\mathrm{O}}_2 $$

FACO_2_ is the fraction of CO_2_ in alveolar gas and FĒCO_2_ is the fraction of CO_2_ in mixed expired gas. In initial studies FĒCO_2_ was obtained by collecting expired gas over time in what is called a Douglas bag with a 60–100 L capacity and then measuring the total volume and the CO_2_ concentration in the bag to calculate FĒCO_2_ [11]. The total volume divided by the time of collection allows calculation of VE.

Bohr’s equation is given more frequently in terms of the partial pressure of CO_2_ instead of fractions:

3 $$ \mathrm{VDphys}/\mathrm{V}\mathrm{T} = \left(\mathrm{P}\mathrm{A}\mathrm{C}{\mathrm{O}}_2\hbox{-}\ \mathrm{P}\bar{\mathrm{E}} \mathrm{C}{\mathrm{O}}_2\right)/\mathrm{P}\mathrm{A}\mathrm{C}{\mathrm{O}}_2 $$

PACO_2_ is alveolar PCO_2_ and PĒCO_2_ is mixed expired PCO_2_ and is measured in the same way as FĒCO_2_.

Partial pressures of CO_2_ are obtained by the following:

4 $$ \mathrm{P}\mathrm{C}{\mathrm{O}}_2 = \mathrm{F}\mathrm{C}{\mathrm{O}}_2 \times \left(\mathrm{PB}\ \hbox{-}\ \mathrm{P}{\mathrm{H}}_2\mathrm{O}\right) $$

PB is barometric pressure and PH_2_O is water vapor pressure. PB equals 760 mmHg and PH_2_O equals 47 mmHg at sea level at normal body temperature.

In an ideal lung with perfect ventilation/perfusion (V/Q) matching, arterial PCO_2_ (PaCO_2_) would be the equivalent of PACO_2_ but V/Q matching is never perfect and PACO_2_ is always less than PaCO_2_. However, PACO_2_ is not readily available whereas PaCO_2_ is. Based on this rationale, in 1938 Enghoff proposed an adaptation of Bohr’s equation in which PaCO_2_ is used instead of PACO_2_:

5 $$ \mathrm{VDphys}/\mathrm{V}\mathrm{T} = \left(\mathrm{P}\mathrm{a}\mathrm{C}{\mathrm{O}}_2\hbox{-}\ \mathrm{P}\bar{\mathrm{E}} \mathrm{C}{\mathrm{O}}_2\right)/\mathrm{P}\mathrm{a}\mathrm{C}{\mathrm{O}}_2 $$

He obtained PaCO_2_ from an arterial blood sample and PĒCO_2_ by the Douglas bag technique [11] as described above.

Severinghaus and Stupfel further demonstrated that changes in VDalv dead space correlate well with changes of arterial to end-tidal CO_2_ gradient (PaCO_2_–ETCO_2_), also called alveolar–arterial CO_2_ difference (A–a CO_2_) [12] and thereby further simplified the dead space calculation. This approximation has become the most popular form of the equation for assessment at the bedside.

## Phases of the volume-based capnogram

Vcap is based on Fowler’s concept. By following changes in expired nitrogen (N_2_) over tidal volume, the respiratory system can be divided in two parts: physiological dead space and effective tidal volume [2]. Bartels et al. [13] showed that expired CO_2_ concentration follows the same curve as that of expired nitrogen (N_2_) after pure oxygen inspiration. Thus, CO_2_ can be substituted for expired N_2_ and plotted against tidal volume. In contrast to the use of N_2_ washout in the Fowler technique, it is not necessary to first ventilate with 100 % O_2_ because the “marker”, which in this case is CO_2_, is already in the alveoli. Use of this approach allows separation of anatomical and alveolar dead spaces on a breath-by-breath basis. Thus, Vcap also is called single-breath test of CO_2_ (SBT-CO_2_).

**Fig. 2:**
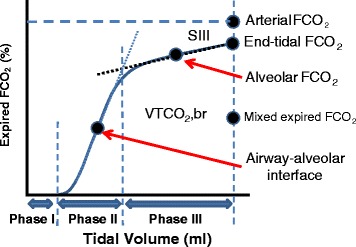
Concentration of CO_2_ during a tidal expiration. Phase I: beginning of expiration; expired gas represents contents of the conduction compartment of the respiratory system. Phase II: transition between anatomic and alveolar dead space. Phase III: alveolar gas. *Expired FCO*_*2*_ (%) fraction of expired CO_2_, *SIII* slope of phase III, *VTCO*_*2*_ *,br* CO_2_ elimination per breath

Figure 2 shows the three phases of SBT-CO_2_. Phase I is from the exhaled tidal volume that is in the airways and not in contact with the alveoli and thus has a negligible concentration of CO_2_. Phase II represents gas coming from regions that are in the transition between anatomic and alveolar gas compartments. This includes gas emptying from small airways and alveoli that are close to the main airways. During this phase there is an almost linear increase in CO_2_. In phase III the slope of expired CO_2_ flattens and plateaus. This phase represents the pure alveolar gas compartment that exists once CO_2_ from the airway–alveolar interface is washed out.

**Fig. 3:**
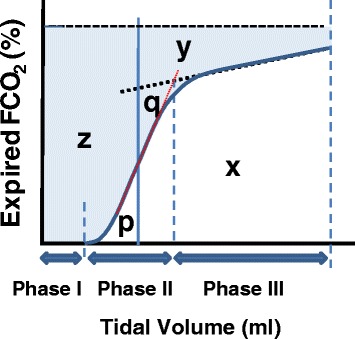
Fletcher approach for evaluating expired gases. The *shaded area* is the total dead space for the breath. Area *z* (area to the left of the *solid line*) is the airway dead space (VDaw), area *y* (area above the slope of phase III) is the alveolar dead space (VDalv, in this case as per Enghoff). As per the Fowler approach [2], area *q* is equal to area *p*. Area *x* (area under capnogram curve) is the volume of CO_2_ expired per breath (VTCO2,br)

Fletcher and colleagues [3] analysis of the plot of expired FCO_2_ against expired tidal volume allowed measurement of the components of dead space on a breath-by-breath basis as shown in Fig. 3. They concluded that PACO_2_ is the midpoint of the line of the slope of phase III, starting at the inflection point (airway-alveolar interface) and terminating at partial pressure of CO2 at the end of expiration (PETCO_2_). This assumption subsequently was validated by Tusman et al. [14], who compared PACO_2_ obtained with Vcap to PACO_2_ obtained with the multiple inert gas elimination technique. The second variable in the Bohr equation is the mixed expired value of PCO_2_ (PĒCO_2_). Values of PĒCO_2_ obtained by Vcap correlate well with values of PĒCO_2_ obtained by other techniques [14–17]. An advantage of the Vcap technique is that it avoids dilution of expired gas from permanent low expiratory flow that is commonly used for triggering the ventilator and thus it provides an accurate measurement of PĒCO_2_ on a breath-by-breath basis. In contrast, the Douglas bag technique overestimates physiological dead space when there is a permanent low expiratory flow in the ventilator circuit [18]. In the example of a Vcap signal in Fig. 3, PĒCO_2_ is measured non-invasively breath-by-breath by taking mean FCO_2_ from the X area. The sum of z and y areas from the graph in Fig. 3 allows calculation of VDphys.

**Fig. 4:**
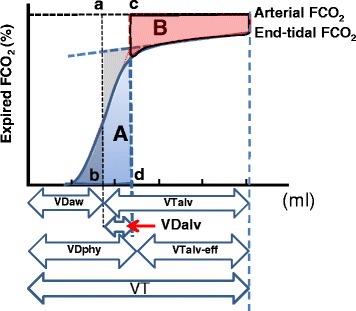
Volumes identified with volumetric capnography (based on Tang et al. [19]). The line a–b defines equal area *q* and *p* as in Fig. 3. The line c–d is created so that area *A* equals area *B*. The distance from *b* to *d* defines alveolar dead space (*VDalv*). Tang et al. did their analysis with the Enghoff approach which uses PaCO_2_ instead of PACO_2_ as in the Bohr approach. If PACO_2_ were used instead the line c–d would be more to the left and the value of VDalv smaller. *VDaw* is the anatomical dead space, *VDalv* is the alveolar dead space, *VDphys* is the physiological dead space, *VTalv-eff* is the efficient alveolar tidal volume, *VTalv* is the alveolar tidal volume, *VT* is the tidal volume

Tang et al. [19] further elaborated on these concepts and used a graphical method to evaluate VDaw, VDalv and VDphys based on classic Vcap. They used an equal area method, which is similar to Fowler’s method for calculating and visualizing physiological and alveolar dead spaces as shown in Fig. 4.

## Difference between Enghoff and Bohr approaches to dead space calculation

**Fig. 5:**
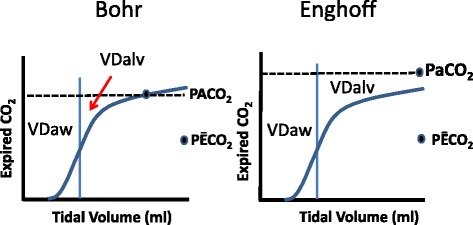
Difference between the Bohr approach and Enghoff approach. *VDaw* is the anatomical dead space, *VDalv* is the alveolar dead space, *PACO*_*2*_ is the alveolar partial pressure of CO_2_, *PaCO*_*2*_ is the arterial partial pressure of CO_2_, *PĒCO*_*2*_ is the mixed expired partial pressure of CO_2_

**Fig. 6:**
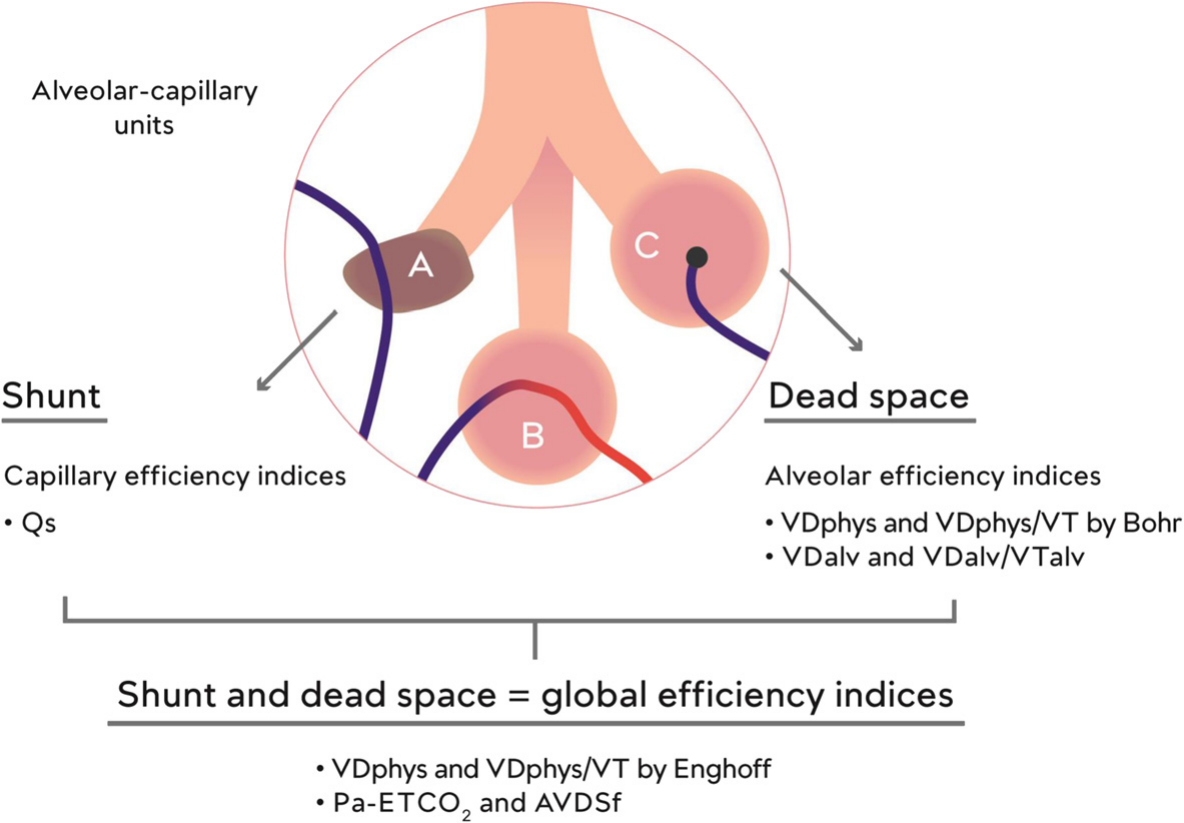
Schematic representation of three-compartment lung model, showing specific indices of capillary, alveolar and global efficiency of gas exchange. See text for abbreviations

As indicated by Tusman et al. [6, 7], substitution of PaCO_2_ for PACO_2_ by Enghoff produces confusion in the interpretation of the mechanisms of dead space production. This substitution is only valid in an ideal lung with perfect V/Q matching for all units, which is never the case. It is especially not true in patients with pulmonary disease and they are a large proportion of the subjects in whom clinicians would want to monitor dead space. The Enghoff substitution also does not allow distinction between an increase in PaCO_2_ due to lack of perfusion of ventilated alveoli, which is true dead space, versus an increase in PaCO_2_ due to CO_2_-enriched blood passing through non-ventilated areas, which is a shunt effect (Fig. 5). The problem is even greater when the arterial to end-tidal CO_2_ (Pa-ETCO_2_) gradient is used as proposed by Severinghaus and Stupfel [12]. However, this limitation of Enghoff’s approach can be put to good use because the difference between the Enghoff- and Bohr-based calculations gives an indication of the shunt component and a global index of pulmonary efficiency and V/Q mismatch [20–22]. The three compartment lung model of Riley shown in Fig. 6 allows visualization of the specific indices of capillary, alveolar and global efficiency of gas exchange.

## Clinical applications of volumetric capnography

Interest in measurement of dead space in acute respiratory distress syndrome (ARDS) patients is based on the study by Nuckton and colleagues [23], who demonstrated that an elevated Enghoff physiological dead space is a strong independent predictor of mortality in early phase ARDS. Their results have been confirmed by using the same technique [24] as well as by Vcap measurement of PĒCO_2_ [25–27] (Table 1). Measurement of dead space could potentially provide a parameter for physicians to follow in early (within 24 hours) or intermediary (within a week) phases of ARDS. Vcap also allows the separation of VDphys from apparent changes in dead space due to shunt and thus can give a more precise indication of physiological mechanisms. Monitoring dead space may aid in the determination of the best PEEP when using increasing or decreasing tidal volumes. This was demonstrated in the classic study by Suter et al. [28] in which “best PEEP” coincided with best pulmonary compliance, which also coincided with lowest Enghoff physiological dead space and maximum oxygen transport.

**Table 1:** Clinical studies of volumetric capnography

	Dead space indices (method)	Clinical impacts	References
ARDS	VDphys/VT (Enghoff approach; equation with PĒCO_2_ estimated by indirect calorimeter or Vcap)	Predictive value of mortality	[23–27]
	VDphys/VT (Enghoff approach; equation with PĒCO_2_ estimated by Douglas bag or Vcap) VDalv, VDalv/VTalv, VAE/VT (Vcap) Pa-ETCO_2_ gradient	Indication values of recruitment with estimation of the best PEEP	[28] Experimental model studies [29, 30]
		Indices of Vcap unmodified during PEEP without recruitment maneuvers	[32, 33]
	VDalv/VT (mainstream CO_2_ sensor)	Indication value of prone position’s response	[36]
	VDphys/VT (Enghoff approach; equation with PĒCO_2_ estimated by indirect calorimeter)	Indices of Vcap unmodified during prone position	[37]
Pulmonary embolism	AVDSf, ETCO_2_/O_2_, time-based capnogram area and Vcap	Diagnostic tool in ER	Meta-analysis [43]
	VDalv/VT (Enghoff approach; Vcap)		[39]
	Fdlate, PE index (Enghoff approach; Vcap)		[40, 42]
	Fdlate, slope III (Enghoff approach; Vcap) Pa-ETCO_2_ gradient AVDSf	Therapeutic efficacy in ER (case report)	[44, 45]
Healthy patient undergoing elective surgery	VDalv/VTalv (Enghoff approach; Vcap)	Indices of Vcap unmodified during prone position	[38]
	VDphys/VT (Enghoff approach; Vcap)	Indication values of recruitment and estimation best PEEP (hysterectomies and hemicolectomies; faciomaxillary surgery)	[46, 47]
Obese patient during bariatric surgery	Slope of phase III (SIII) (Vcap)	Indication values of recruitment and estimation of the best PEEP	[48]
	VTCO_2_,br and VDphys/VT (Bohr approach; Vcap)		[49]
One-lung ventilation during thoracic surgery	VDalv/VTalv (Enghoff approach; Vcap)	Indication values of recruitment and estimation of the best PEEP	[51]
	VDalv/VTalv, VDphys/VT (Enghoff approach; Vcap)	Physiological dead space did not change but alveolar indice of Vcap improved during recruitment	[52]
Weaning from ventilator	VDphys/VT (Enghoff’ approach; Vcap)	Predictive value of successful extubation (pediatric and adult population)	[53, 54]

*AVDSf* alveolar dead space fraction, *ER* emergency room, *Fdlate* late dead space fraction, *PE index* ratio between PaCO_2_-ETCO_2_ and slope of phase III’s plateau, *PEEP* positive end-expiratory pressure, *Vcap* volumetric capnography

In a model of lung injury in pigs, Tusman et al. [29] showed that VDalv, VDalv/alveolar tidal volume (VTalv) obtained by Vcap (Enghoff approach) and Pa-ETCO_2_ gradient are sensitive and specific indicators of the lung’s efficiency of gas-exchange during PEEP titration conducted after a recruitment maneuver (RM). More recently, Tusman et al. [30] used the same lung injury model to compare Vcap measurements with the multiple inert gas elimination technique. The lowest VDalv/VTalv and slope of phase III (SIII) obtained with Vcap by the Enghoff analysis (i.e., use of PaCO_2_) and Pa-ETCO_2_ gradient were associated with lung recruitment during PEEP titration [30]. SIII is an index of V/Q mismatching; a decrease in SIII corresponds to improved V/Q homogeneity [31].

In contrast to these studies, Blanch et al. [32] found no correlation between level of PEEP (from 0–15 cmH2O) and VDphys/VT measured by Vcap in both healthy and ARDS subjects, although Vcap indices correlated with disease severity. However, one would not have expected any change in Vcap measurements in their study because lung recruitment maneuvers were not used to open lung units and there was no change in static compliance, indicating that there was no lung recruitment with PEEP to alter the measurements. Furthermore, the results were presented as group data, which may have masked changes in individuals who actually had lung recruitment. The same likely is true for the study by Beydon et al. [33] who also found no change in VDalv/VT measured by Vcap with increasing of PEEP in ARDS patients.

Prone position redistributes perfusion from posterior to anterior lung units due to the force of gravity and recruitment of posterior lung units improves V/Q homogeneity, which improves oxygenation and reduces PaCO_2_ [34, 35]. Gattinoni et al. [35] showed that subjects who had evidence of a decreased VDphys in the prone position based on a decrease in PaCO_2_ had lower mortality (mortality at day 28 was 35.1 % versus 52.2 %, relative risk = 1.48 with confidence intervals 1.07–2.05, *p* = 0.01) [35]. Charron et al. [36] showed that Pa-ETCO_2_/arterial partial pressure of oxygen (PaCO_2_) decreases in the prone position and concluded that change in PaCO_2_ is a better indicator of a positive response to prone positioning than changes in PaO_2_/FiO_2_ (fraction of inspired O_2_). However, other investigators have found no significant change in dead space during prone positioning in ARDS patients [37] and healthy patients undergoing long duration elective surgery [38].

## Pulmonary embolism

The presence of positive D-dimers has a sensitivity of 93.8 % and specificity of 67 % for diagnosis of pulmonary embolism (PE) [39]. Thus, a negative D-dimer makes the diagnosis of a PE unlikely, especially if the pre-test probability is low or moderate. Kline et al. showed that if Vcap-derived alveolar dead space fraction (VDalv/VT) is less than 20 %, and the D-dimers test is negative, the sensitivity of detecting a PE is increased to 98.4 % [39] and there is little value for the use of further tests in patients with low pretest probability of PE.

Vcap also can be used to exclude pulmonary embolism when D-dimer concentrations are positive. Verschuren et al. [40] compared Vcap with PaCO_2_-ETCO_2_ gradient in patients suspected of having PE and who had positive D-dimers. They evaluated a number of derived values from the Vcap plot and found that the best indicator was an elevation of what they called "late dead space fraction" (Fdlate), which is defined as (PaCO_2_-expCO_2_ at 15 % of total lung capacity)/PaCO_2_. Eriksson et al. [41], too, found that a Fdlate >12 % was a good predictor of PE. More recently, Verschuren et al. [42] used their same Vcap data set to compare the diagnostic value for the prediction of PE based on alveolar dead space fraction (AVDSf), which is defined as (PaCO_2_-ETCO_2_)/PaCO_2_, to Fdlate and pulmonary embolism index (PE index), which is defined as (PaCO_2_-ETCO_2_)/slope of phase III. The Vcap measures were not superior (using AVDSf of less than 15 %) for the exclusion of PE in outpatients with low clinical probability and positive D-dimer test results. However, they did not calculate PACO_2_ and thus could not measure alveolar dead space, which likely would be the more defining value obtained from Vcap. In a meta-analysis [43] of 14 trials with a total of 2291 patients and an average prevalence of PE of 20 % the authors concluded that capnography had good sensitivity (80 %) but low specificity (49 %). Specifically, in the subgroup of patients with a low-probability of PE (Wells score < 2), a negative test excludes a PE even when D-dimers are positive. The major limitation of this analysis is that 12 of the 14 studies were time-based and only two were volumetric-based and thus Vcap measures were greatly undervalued.

Vcap also can be used for bedside monitoring of the efficacy of thrombolysis in patients with major pulmonary embolism [44, 45] by directly following change in VD fraction on a breath-by-breath basis during thrombolysis and looking for a decrease in VDalv/VT.

In summary, a simple alveolar dead space fraction (AVDSf), or ideally Vcap, in the emergency room could potentially decrease the number of contrast computed tomographic chest studies or V/Q lung scans in patients with positive D-dimers and low pretest probability of PE. They also can be used to track the efficacy of therapy. Further studies are needed in the emergency room to establish the safety profile of Vcap. It also will be important to determine if failure to find improved diagnostic efficacy with Vcap in previous studies was because of use of the Enghoff rather than the Bohr approach.

## Others applications of Vcap

Vcap can be helpful for monitoring the response to titration of PEEP. As shown by Suter et al. [28], optimal PEEP should provide not only best oxygenation and compliance, but also the lowest VD. By exploring both sides of the alveolar–capillary barrier at the bedside, Vcap can be useful for avoiding atelectasis and opening-injury during an upward and downward PEEP titration procedure, which could help to reduce ventilator-induced lung injury. Vcap has been used during elective surgery in healthy patients to monitor recruitment in an attempt to obtain the lowest VD and highest compliance [46, 47]. For example, Bohm et al. used Vcap in morbidly obese patients to show that the slope of phase III decreases with recruitment maneuvers during bariatric laparoscopic surgery [48]. Tusman et al. [49] showed that the highest pulse oximetry oxygen saturation (SpO_2_), the lowest VD and the highest VTCO_2_,br (CO_2_ production per breath) occur at the PEEP level that keeps alveoli open. They also studied patients undergoing one-lung ventilation (OLV) during thoracic surgery [50]. In thoracic surgery one of the main goals of anesthesiologists is to maintain adequate minute ventilation while keeping tidal volume below a level that over-distends dependent lung units and produces VD and lung injury. However, the consequence of this strategy is hypercapnia and respiratory acidosis. In a randomized controlled trial at a single center, use of Vcap to guide alveolar recruitment before and after OLV reduced alveolar dead space (Enghoff’s approach) and increased oxygenation without changing dynamic compliance [51]. Ferrando et al. [52] observed that the optimal PEEP, which corresponded to the maximal dynamic compliance after a recruitment maneuver during OLV (study group), maintained better oxygenation and better static compliance without a change in VD fraction (Vcap with Enghoff approach). This may have been because the control group also benefited from recruitment manoeuvers and PEEP 5 cmH_2_O [52].

Vcap measurements may also help wean patients from the ventilator. Hubble et al. [53] observed that successfully extubated children had lower physiological VD fractions (Vcap using Enghoff approach). A VD fraction ≤ 0.5 was associated with 96 % success of extubation. These findings were confirmed in a mixed adult population [54]. In 77.6 % of successfully extubated patients the VD fraction was <0.5.

## Non-invasive measurement of cardiac output

Cardiac output can be estimated non-invasively based on the Fick equation:

6 $$ \mathrm{Q} = \mathrm{V}\mathrm{C}{\mathrm{O}}_2/\left(\mathrm{C}\mathrm{v}\mathrm{C}{\mathrm{O}}_2\hbox{-}\ \mathrm{C}\mathrm{a}\mathrm{C}{\mathrm{O}}_2\right) $$

Q is pulmonary capillary blood flow, VCO_2_ is CO_2_ elimination per minute, CvCO_2_ is CO_2_ content of mixed venous blood and CaCO_2_ is CO_2_ content of arterial blood. If minute ventilation and cellular metabolism are stable, changes in VCO_2_ measured by Vcap should parallel changes in pulmonary capillary blood flow as would be expected following a fluid challenge. An automated capnodynamic method that measures cyclic changes of PETCO_2_ and CO_2_ elimination rate from cyclic changes in tidal volume allows for on-line monitoring of effective pulmonary capillary blood flow [55]. The ability of a change in continuous VCO_2_ followed by Vcap to predict a decrease in cardiac output after an increase in PEEP recently was demonstrated in patients undergoing cardiac surgery [56].

## Conclusion

Vcap allows precise measurement of physiological and alveolar dead spaces on a breath-by-breath basis at the bedside. It thereby allows quantification of global V/Q mismatches and allows separation of the components such as true dead space on the alveolar side of the alveolar–capillary membrane and shunt on the capillary side. The most promising Vcap parameters are (i) physiological dead space fraction (VDphys/VT) based on the Enghoff approach (this gives a global index of V/Q mismatch including shunts and low V/Q areas); (ii) physiological dead space based on the Bohr approach (VDphys/VT); and (iii) alveolar dead space fraction (VDalv/VTalv). The last two represent true indices of lung efficiency at the alveolar side of the alveolo–capillary membrane. Differences between the Bohr and Enghoff approaches may provide the most useful information. Vcap is a promising tool that is based on physiological concepts. Further research is needed to define its diagnostic value and potential utility for guiding therapy of patients in the emergency department, operating room and intensive care unit.

## Abbreviations

ARDS, acute respiratory distress syndrome; AVDSf, alveolar dead space fraction; CaCO_2_, arterial CO_2_ content; CO_2_, carbon dioxide; CvCO_2_, mixed venous CO_2_ content; ETCO_2_, end-tidal CO_2_ in expired gas; FACO_2_, fraction of CO_2_ in alveoli; Fdlate, (PaCO_2_-expCO_2_ at 15 % of total lung capacity)/PaCO_2_; FĒCO_2_, fraction of CO_2_ in mixed expired gas; FiO_2_, fraction of inspired O_2_; N2, nitrogen; OLV, one-lung ventilation; PACO_2_, alveolar PCO_2_; PaCO_2_, arterial PCO_2_; PB, barometric pressure; PCO_2_, partial pressure of carbon dioxide; PE, pulmonary embolism; PĒCO_2_, partial pressure of CO_2_ in mixed expired gas; PEEP, positive end-expiratory pressure; PETCO_2_, partial pressure of CO2 at the end of expiration; PH_2_O, partial pressure of water vapour; SBT, single breath test; V/Q, ventilation perfusion ratio; VA, alveolar ventilation; Vcap, volumetric capnography; VCO_2_, CO_2_ elimination per minute; VD, ventilation dead space; VDalv, alveolar dead space; VDaw, dead space in conducting airways; VDinst, dead space ventilation in the instrument; VDphys, physiological dead space; VE, total minute ventilation; VT, tidal volume; VTalv, alveolar tidal volume; VTCO_2_,br, CO_2_ production per breath
